# Racial and Ethnic Disparities in the Diagnosis and Treatment of Obstructive Sleep Apnea: A Systematic Review

**DOI:** 10.3390/ijerph23050614

**Published:** 2026-05-05

**Authors:** Siji Thomas, Shafer G. Tharrington, Aditya Patel, Mevelyn Kaalla, Adarsh Thomas, Nikhil Madala, Younghoon Kwon, William J. Healy

**Affiliations:** 1 Department of Pulmonary Medicine, Augusta University, Augusta, GA 30912, USA; nmadala@augusta.edu (N.M.); wihealy@augusta.edu (W.J.H.); 2 Department of Allied Health Sciences, Augusta University, Augusta, GA 30912, USA; stharrington@augusta.edu; 3 Medical College of Georgia, Augusta University, Augusta, GA 30912, USA; adipatel@augusta.edu; 4 Department of Internal Medicine, Medical College of Georgia, Augusta, GA 30912, USA; mkaalla@augusta.edu; 5 Biomedical Sciences, Texas A&M University, College Station, TX 77843, USA; adarshsthomas05@tamu.edu; 6 Division of Cardiology, University of Washington, Seattle, WA 98195, USA; yhkwon@uw.edu

**Keywords:** health disparities among minority and vulnerable populations, ethnic and racial minorities, sleep apnea diagnosis and treatment, sleep apnea among minority groups

## Abstract

**Highlights:**

**Public health relevance—How does this work relate to a public health issue?**

- Untreated sleep apnea can impair neuro- and cardiovascular health.
- Lack of timely diagnosis and treatment can increase global health care utilization costs.

**Public health significance—Why is this work of significance to public health?**

- This paper highlights the impact of the underdiagnosis and undertreatment of sleep apnea among racial and ethnic minorities.
- Early diagnosis and treatment of sleep apnea will increase quality of life and life expectancy among minority populations globally.

**Public health implications—What are the key implications or messages for practitioners, policymakers, and/or researchers in public health?**

- Addressing the root causes of impaired sleep health and inadequate access to diagnosis and treatment requires a multimodal approach.
- Sleep apnea phenotyping may be required when personalizing treatment strategies in different communities.

**Abstract:**

Background: Sleep apnea is identified and treated less frequently among racial and ethnic minorities. Objective: The purpose of this systematic review was to examine disparities among racial and ethnic minorities and to understand the reasons for poor sleep health. Methods: The authors conducted a literature search using PubMed and Cochrane Library databases, last accessed in September 2025, using regular and MeSH keywords. A total of 123 articles were identified. PRISMA guidelines were followed, the PICO framework was applied, and the inclusion criteria were based on studies conducted in the past 10 years. After quality assessment, 18 studies were included for in-depth analysis. Results: The 18 studies included meta-analyses and observational cohort studies. In total, 51,489 patients were represented. Studies revealed that sleep apnea is underdiagnosed and undertreated in ethnic minority populations. Resident location, gender, economic status, and marital status also play an important role. One study noted clinically insignificant differences in positive airway pressure requirements between black and white populations. Nocturnal hypertension and increased left ventricle size are also observed in untreated sleep apnea. Given the heterogenous nature of the studies, quality risk assessment was not possible, which is a limitation of this study. Conclusions: Sleep apnea is underdiagnosed and undertreated among ethnic minorities. Factors such as ancestry, comorbidities, social determinants, geography, and healthcare access drive global inequities. Further sleep apnea phenotyping may be of value in planning treatment strategies.

## 1. Introduction

Sleep apnea is a common disease that is now more widely recognized because of the rise in prevalence and major neurocognitive and cardiovascular consequences, but available data suggest that most cases of obstructive sleep apnea remain undiagnosed and untreated, even in developed countries [1,2]. Obstructive sleep apnea is associated with an elevated risk of hypertension and cardiovascular disease [3]. It is known that rates of cardiovascular morbidity and mortality remain high among racial and ethnic minorities. The incidence of sleep apnea is also reportedly high among minority groups; however, timely diagnosis and treatment of sleep apnea remain infrequent in these populations [4,5]. Given the dearth of studies exploring these differences globally, a systematic review was done to examine disparities specifically among racial and ethnic minorities and to understand the factors preventing accurate diagnosis and treatment of sleep apnea in these populations. We also aim to understand the root cause of poor sleep health to prevent cardiovascular morbidity and mortality among racial and ethnic minorities. We anticipate that this study will fill the gap between the underlying causes of poor sleep health and implementing interventions geared towards addressing these deficiencies to improve sleep health in vulnerable populations internationally.

## 2. Materials and Methods

Using regular and MESH terms, we conducted a literature search for randomized controlled trials, systematic reviews, meta-analyses, and observational cohort studies. The databases searched were PubMed and Cochrane Library, and the last date of the search was 9 May 2025. The types of studies included reviews, protocols, and trials. The mesh terms used included “Adults”, “Minority Groups”, “Ethnic and Racial Minorities”, “Health Disparate Minority and Vulnerable Populations”, “Sleep Apnea”, “Obstructive”, “Apnea”, “Continuous Positive Airway Pressure“, “Polysomnography”, “Sleep study”, “Early Diagnosis”, “Diagnosis”, “Cardiovascular Diseases”, “Cardiovascular System”, and “Apnea-Hypopnea Index” OR “Apnea-Hypopnea Index” OR “AHI”. The search included English-language studies published worldwide in the past 10 years that explored the populations of interest and sleep apnea treatment. Multiple study designs were included, such as observational, experimental, and critical review studies. Heterogenous studies were included to address both diagnosis and treatment aspects of sleep apnea. Studies were organized to reflect diagnostic methodology and available treatment modalities. Articles published before 2015 were excluded. Two separate researchers were engaged in data extraction, and generative artificial intelligence was not used in any phase of the research.

## 3. Results

### 3.1. Research Results

**Figure 1:**
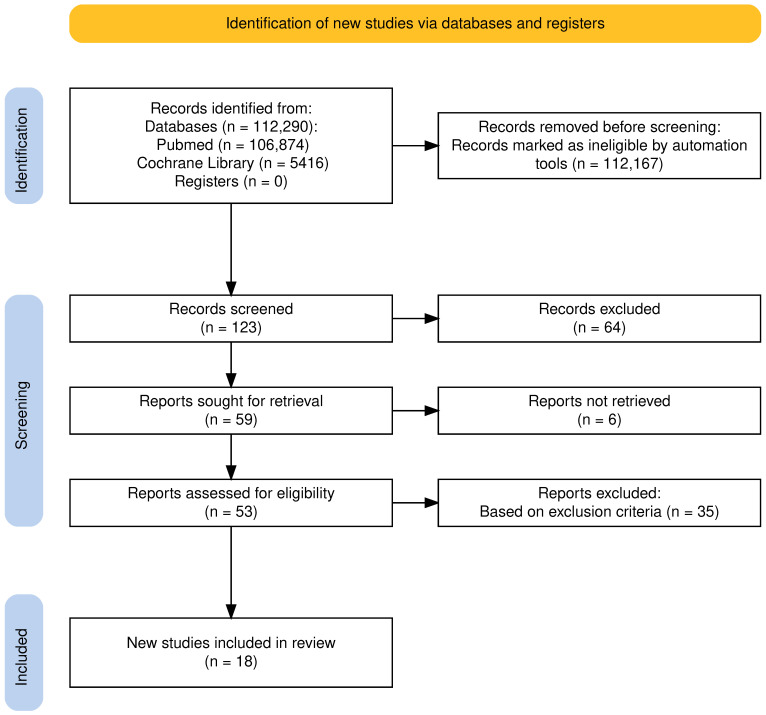
PRISMA flow diagram illustrating the study selection and literature inclusion process. PRISMA: Preferred Reporting Items for Systematic Reviews and Meta-Analyses [6].

Among the 112,290 articles searched, initial screening yielded 123 articles. Duplicates and irrelevant articles were excluded using EndNote, after which 59 articles were retrieved, and 64 reports were omitted due to irrelevancy. The final screening reduced the number of reports to 53, all of which were evaluated for quality and eligibility. After thorough reading, 18 eligible reports were included in this study. Two researchers independently extracted and identified data from each study and used the appropriate quality assessment techniques to examine each study’s efficacy. When there were differences of opinion, the two researchers considered the study designs, inclusion and exclusion criteria, interventions used, and outcome evaluation to reach a consensus. In ambiguous instances, a third author was brought in to settle disagreements and reach a consensus. Figure 1 depicts the search process used for this review in the form of a PRISMA flow diagram. The PRISMA Checklist can be found in File S1. The studies included were observational studies, including cross-sectional studies, systematic reviews and epidemiological studies.

The characteristics of the studies, including the type, year of publication, and findings, are provided in Table 1 below [7,8,9,10,11,12,13,14,15,16,17,18,19,20,21,22,23,24].

### 3.2. Study Characteristics

The studies included observational studies, meta-analyses, systematic reviews, quasi-experimental, and cross-sectional studies. This review covered a collective sample of more than 50,000 studies. Highlights and limitations of the studies are also included in the tables. One study used admixture mapping techniques. Attempts were made to include both national and international studies. Attempts have been made to include the global population and minority sections from all over the world. Table 1 describes the type of study, population included, outcomes, and limitations of individual studies.

### 3.3. Sleep Apnea Diagnosis

Most studies included in this review examined racial disparities in sleep apnea diagnoses. One study revealed that published sleep studies in general do not address the representativeness of patients undergoing sleep studies in a community setting.

### 3.4. Sleep Apnea Treatment

About five studies addressed sleep apnea treatment, specifically CPAP use. These studies investigated treatment compliance, mortality benefit, and CPAP requirements. Collectively, they included around 13,000 patients.

## 4. Discussion

Obstructive sleep apnea (OSA) is one of the most prevalent pulmonary disorders related to sleep. It consists of episodes of apnea (complete) or hypopnea (partial) due to upper airway obstruction, which ultimately disrupts sleep latency and decreases oxygen saturation levels. OSA can lead to decreased sleep, fatigue, and overall poor quality of life. If left untreated, it can lead to an increased rate of cardiovascular disease, stroke, metabolic disease, excessive daytime sleepiness, workplace errors, traffic accidents, and death. Untreated OSA is also associated with perioperative complications, such as prolonged intubation, need for re-intubation, pneumonia, aspiration, arrhythmia, and cardiac arrest [25]. Therefore, early diagnosis and treatment of OSA is crucial to increasing the quality of life and overall life expectancy of afflicted individuals.

It is known that OSA is more prevalent among men than women. In the United States, one study found that among adults 30–70 years of age, approximately 13% of men and 6% of women had moderate to severe sleep disordered breathing (apnea–hypopnea index ≥15), fitting the Medicare criteria for a positive indication of OSA [26,27]. Are there Ethnic and racial disparities when it comes to the diagnosis and treatment of OSA? Is there any evidence of increased prevalence of OSA among minorities and vulnerable individuals? Since we have included global studies, we do not attempt to define minorities and vulnerable groups in this review, as they differ in different regions of the globe, and leave it up to the readers to make this distinction. Although few studies have investigated OSA and its association with race, there is strong evidence to suggest that African Americans face poorer outcomes. One study showed that African Americans had a similar prevalence of OSA as white participants (32% and 30%, respectively) within the age group of 65 years and older; however, this group was 2.1 times more likely to have severe OSA [28]. Compared to the Non-Hispanic White group, the Hispanic and Asian/Pacific Islander groups were 1.45 and 1.81 times more likely to have moderate to severe OSA, respectively [29]. Similarly, moderate to severe OSA was 1.7 times more common among Native American participants than White participants [30]. In contrast, Asian American patients have a similar or lower prevalence compared to White patients. A study focusing on the Japanese population demonstrated that the prevalence of sleep-disordered breathing was higher in Hispanic (36.5%) and White (33.3%) individuals than among Japanese individuals (18.4%), corresponding to differences in body mass index [31]. However, other studies showed similar degrees of OSA severity among these ethnic groups [32]. This study was conducted to provide a systematic review of racial disparities in the diagnosis and treatment of obstructive sleep apnea to improve outcomes for vulnerable patient populations and understand the root causes of these disparities.

Across the 18 studies reviewed, there was significant variability in the racial, ethnic, gender, and age representation of study populations. White and Asian participants were most frequently included in OSA research, while Black and Hispanic/Latino groups were often underrepresented, especially in surgical cohorts for which race was inconsistently reported or not reported at all [9,14]. Several studies focused exclusively on minority populations, such as Aboriginal Australians [13] or African Americans [15,19,33]. This helped highlight the unique challenges and risk profiles these groups face, but it also underscored how rare these focused efforts are. Gender disparities were also prominent. In surgical studies, the participants were overwhelmingly male (83%), despite the OSA prevalence being roughly two-to-one in men versus women [14]. In contrast, diagnostic studies involving minority populations often had a female majority, such as the urban African American cohort in Pittsburgh, in which nearly 80% of participants were women. This study, the PHRESH trial, was another landmark trial that objectively examined urban patients [16]. Age distributions varied by region, with community cohorts like MESA skewing older (mean age ~68 years) [20,23] and Aboriginal Australian studies including younger, working-age adults [13]. Collectively, these patterns reveal critical gaps in representation in OSA research, limiting the generalizability of findings and highlighting the need for more inclusive study designs.

When examining how OSA presentation and outcomes differ globally, clear geographic and racial disparities emerged. In the U.S., African American adults consistently exhibited higher rates of undiagnosed OSA and more severe nocturnal hypoxemia, particularly among women and those with obesity. The Jackson heart study, like the Cardia trial, examined the association between the development of hypertension and nocturnal hypoxemia [7,15,19]. Despite elevated risk for these conditions, African American patients were less likely to receive or adhere to Continuous Positive Airway Pressure (CPAP) therapy compared to White patients, even in settings with equivalent access, further emphasizing structural and cultural barriers [17,22]. Wang and colleagues carried out a landmark admixture study, the first of its kind, with a large cohort of 11,575 participants. Their study attempted to shed light on genetic variations among different ethnic groups [18] and found that Hispanic adults presented with distinct challenges, a subject that also appears to be less well studied. Wang et al. [34] demonstrated that ancestry substructure within Hispanic/Latino populations influenced OSA risk, while Vazquez et al. [12] found that Hispanic patients had more severe OSA despite being younger and faced higher rates of missed follow-up care. National data confirmed that Hispanic adults were significantly less likely to receive CPAP treatment than White adults, even after adjusting for socioeconomic factors [34]. Outside the U.S., Aboriginal Australians experienced unique obstacles to OSA care. Aboriginal Australians in remote communities demonstrated much lower adherence to CPAP due to factors such as geographic isolation, inconsistent electricity, and cultural perceptions of treatment [13]. Finally, a Canadian study involving homeless shelter residents illustrated how socio-economic barriers further exacerbate diagnostic disparities and underscored the need for innovative, accessible screening tools [34]. These findings emphasize that both biological factors, such as ancestry and comorbidities, and social determinants, including geography and healthcare access, drive global inequities in OSA care. Interestingly, a prospective study was conducted to assess the influence of perceived racial discrimination on CPAP usage and found that adults who encountered racial discrimination experienced a greater decline in CPAP usage over time [35]. Our study is not without limitations. We found no randomized control trials that could be included, and heterogeneity and a lack of quality assessment methods for certain types of study posed difficulties. Furthermore, the lack of inclusion of prospective studies can be considered a drawback of our review. However, we attempted to investigate a large, heterogenous, multiethnic population from studies published within the last ten years. Future multicentric prospective studies on sleep phenotype may be of value. Phenotype-based intervention might help address the root cause of these disparities in the future.

## 5. Conclusions

Poor sleep health exists among racial and ethnic minorities globally, and the reasons for inequity in sleep healthcare are multifactorial. Biological factors, such as ancestry and comorbidities, and social determinants, including geography and healthcare access, drive global inequities in sleep health management. Large-scale global awareness and education are needed. Further sleep apnea phenotyping may be of value in planning treatment strategies, and future multicentric prospective studies could be beneficial.

## Figures and Tables

**Table 1:** Details of the studies included.

Sr No.	Names of Authors	Title	Type of Study	Journal/Year	Sample Size	Results/Outcome	Highlights/Limitations
1 [7]	Leng, Y., Cavaillès, C., Peltz, C., O’Bryant, S.E., Redline, S., Yaffe, K.	Racial and Ethnic and Sex Differences in At-Home Estimates of Obstructive Sleep Apnea Parameters among Diverse Adults	Observational Study	*Ann. Am. Thorac Soc.* 2025	821	More REM-stage respiratory events occur in Black adults, particularly Black women, compared with their non-Hispanic white counterparts.	Objective study; attempted to phenotype sleep apnea.
2 [8]	Goosmann, M., Williams, A.M., Springer, K., Yaremchuk, K.L.	The Impact of Marital Status and Race in Obstructive Sleep Apnea	Observational Study, Retrospective	*Ear Nose Throat J.* 2025	6200	Married patients with OSA had increased survival compared to their single counterparts. Married Black patients had the highest survival.	Large population, only included married black and white races in the study
3 [9]	Nanu, D.P., Diemer, T.J., Nguyen, S.A., Tremont, T., Meyer, T.A., Abdelwahab, M.	Racial variations in maxillomandibular advancement for obstructive sleep apnea: A systematic review and meta-analysis	Meta Analysis	*Sleep Breath.* 2024	469	Asian patients tend to have more severe OSA preoperatively and experience greater postoperative improvements in AHI, LSAT, and ESS compared to Caucasians. There is a racial disparity in MMA outcomes amongst Black and Latino patients, with a lack of studies on these groups.	Most studies were case series. None included black patients.
4 [10]	Wang, V.H., Li, Y., Kent, D.T., Pagán, J.A., Arabadjian, M., Divers, J., Zhang, D.	Racial and ethnic differences in the receipt of continuous positive airway pressure treatment for obstructive sleep apnea	Cross-Sectional Study	*Sleep Med.* 2024	8518	The rates of CPAP treatment among OSA patients are not consistent across racial and ethnic groups; Hispanic patients are less likely to receive CPAP treatments.	Used a national database with a large sample size and wide geographic coverage to assess disparities.
5 [11]	Cai, Y., Parekh, M.H., Rodin, J., Tangutur, A., Yu, J.L., Keenan, B.T., Schwartz, A.R., Dedhia, R.C.	Differences in Positive Airway Pressure Requirements in Obstructive Sleep Apnea Between Black and White Patients	Retrospective Study	*Otolaryngol. Head Neck Surg.* 2024	391	Black and White OSA patients had clinically insignificant differences in PAP requirements.	Black and White patients may have comparable PAP alternative responses from a collapsibility standpoint; a statistically significantly higher percentage of Black patients are excluded (49.7%); good-quality CPAP data.
6 [12]	Vazquez, I.M., Park, M., Ferri, R., Mogavero, M.P., DelRosso, L.M.	Sleep and follow-up characteristics of Hispanic patients: Insights from a comparative analysis with White patients in polysomnographic split-night studies	Observational Retrospective study	*Sleep Med.* 2024	95	Hispanic population exhibited significantly worse OSA parameters during the diagnostic phase of PSG compared to White patients.	Study attempted to compare sleep parameters in split studies; Hispanic patients had a significantly higher no-show percentage.
7 [13]	Heraganahally, S.S., Howarth, T.P., Perez, A.J., Crespo, J., Atos, C.B., Cluney, B.J., Ford, L.P.	Acceptability, adaptability and adherence to CPAP therapy among Aboriginal Australians with OSA “The A5 study”	Observational Retrospective study	*Sleep Med.* 2023	649	Long-term adherence to CPAP therapy was significantly influenced by patients’ residential location, with those in remote/rural settings demonstrating significantly lower rates.	The Australian Aboriginal study is less generalizable.
8 [14]	Debbaneh, P., Ramirez, K., Block-Wheeler, N., Durr, M.	Representation of Race and Sex in Sleep Surgery Studies	Systematic Review	*Otolaryngol. Head Neck Surg.* 2022	13,078	There is a racial/ethnic and sex inclusion bias among sleep surgery studies.	Study calls for better external validity of sleep research in terms of standard of representativeness.
9 [15]	Thomas, S.J., Johnson, D.A., Guo, N., Abdalla, M., Booth, J.N., Spruill, T.M., Jackson, C.L., Yano, Y., Sims, M., Calhoun, D., Muntner, P., Redline, S.	Association of Obstructive Sleep Apnea with Nighttime Blood Pressure in African Americans: The Jackson Heart Study	Observational Epidemiological Study	*Am. J. Hypertens.* 2020	206	Severity of OSA and nocturnal hypoxemia were associated with high nighttime BP in African American participants in the Jackson Heart Study.	Data on ambulatory BP and sleep were collected in a large community-based sample of African Americans following a standardized protocol. Less generalizable but similar findings were obtained in the CARDIA study.
10 [16]	Dong, L., Dubowitz, T., Haas, A., Ghosh-Dastidar, M., Holliday, S.B., Buysse, D.J., Hale, L., Gary-Webb, T.L., Troxel, W.M.	Prevalence and correlates of obstructive sleep apnea in urban-dwelling, low-income, predominantly African American women	Longitudinal Quasi-Experimental Study	*Sleep Med.* 2020	828	Low-income African Americans, including women, are a high-risk group for OSA but remain under-diagnosed and under-treated.	First study to objectively measure neighborhood walkability and OSA in urban African American adults; PHRESH study.
11 [17]	Quintos, A., Naranjo, M., Kelly, C., Quan, S.F., Sharma, S.	Recognition and Treatment of Sleep-disordered Breathing in Obese African American Hospitalized Patients May Improve Outcome	Post Hoc Analysis	*J. Natl. Med. Assoc.* 2019	2022	This large study confirms a high prevalence and lower adherence to PAP therapy among African Americans. Adherent patients, however, showed a mortality benefit like Caucasians.	Post hoc analysis from HoSMed database.
12 [18]	Wang, H., Cade, B.E., Sofer, T., Sands, S.A., Chen, H., Browning, S.R., Stilp, A.M., Louie, T.L., Thornton, T.A., Johnson, W.C., Below, J.E., Conomos, M.P., Evans, D.S., Gharib, S.A., Guo, X., Wood, A.C., Mei, H., Yaffe, K., Loredo, J.S., Ramos, A.R., Barrett-Connor, E., Ancoli-Israel, S., Zee, P.C., Arens, R., Shah, N.A., Taylor, K.D., Tranah, G.J., Stone, K.L., Hanis, C.L., Wilson, J.G., Gottlieb, D.J., Patel, S.R., Rice, K., Post, W.S., Rotter, J.I., Sunyaev, S.R., Cai, J., Lin, X., Purcell, S.M., Laurie, C.C., Saxena, R., Redline, S., Zhu, X.	Admixture mapping identifies novel loci for obstructive sleep apnea in Hispanic/Latino Americans	Admixture Mapping study	*Hum. Mol. Genet.* 2019	11,575	Local African ancestry at the chromosomal region 2q37 genome-wide was significantly associated with AHI (*p* < 5.7 × 10^−5^); European and Amerindian ancestries at 18q21 were potentially associated with both AHI and percentage time SaO_2_ < 90% (*p* < 10^−3^).	First admixture mapping analysis of OSA phenotypes; largest cohort of individuals with Hispanic/Latino background.
13 [19]	Johnson, D.A., Guo, N., Rueschman, M., Wang, R., Wilson, J.G., Redline, S.	Prevalence and correlates of obstructive sleep apnea among African Americans: The Jackson Heart Sleep Study	Longitudinal Study	*Sleep* 2018	852	High prevalence of OSA among a large population of African American men and women; the majority (95%) were undiagnosed and untreated.	Neither sleepiness nor waist circumference was associated with OSA in this population.
14 [20]	Geovanini, G.R., Wang, R., Weng, J., Jenny, N.S., Shea, S., Allison, M., Libby, P., Redline, S.	Association between Obstructive Sleep Apnea and Cardiovascular Risk Factors: Variation by Age, Sex, and Race. The Multi-Ethnic Study of Atherosclerosis	Cross-Sectional Study	*Ann. Am. Thorac Soc.* 2018	1344	Several cardiovascular risk associations were stronger in men, younger individuals, and African American individuals.	A multi-ethnic study.
15 [21]	Piovezan, R.D., Hirotsu, C., Feres, M.C., Cintra, F.D., Andersen, M.L., Tufik, S., Poyares, D.	Obstructive sleep apnea and objective short sleep duration are independently associated with the risk of serum vitamin D deficiency	Cross-Sectional Study	*PLoS ONE* 2017	657	OSA and short sleep duration showed significant independent associations with the risk of 25(OH)D deficiency, affecting more female individuals, obese individuals, African Americans, smokers, sedentary patients, and those with hypertension and DM.	Study performed in the city of Sao Paulo, Brazil.
16 [22]	Schwartz, S.W., Sebastião, Y., Rosas, J., Iannacone, M.R., Foulis, P.R., Anderson, W.M.	Racial disparity in adherence to positive airway pressure among US veterans	Retrospective Observational study	*Sleep Breath.* 2016	2172	CPAP compliance is considerably lower in AA patients than White patients.	VA study.
17 [23]	Javaheri, S., Sharma, R.K., Wang, R., Weng, J., Rosen, B.D., Bluemke, D.A. Lima, J.A., Redline, S.	Association between Obstructive Sleep Apnea and Left Ventricular Structure by Age and Gender: The Multi-Ethnic Study of Atherosclerosis	Cross-Sectional Study	*Sleep* 2016	1412	LV mass was significantly increased with increasing AHI category for subjects aged 65 y or younger. The association between the AHI and LV mass was stronger in white and Chinese patients compared to black and Hispanic patients, although the difference was not statistically significant.	Community-based ethnically diverse study.
18 [24]	Woods, C.E., McPherson, K., Tikoft, E., Usher, K., Hosseini, F., Ferns, J., Jersmann, H., Antic, R., Maguire, G.P.	Sleep Disorders in Aboriginal and Torres Strait Islander People and Residents of Regional and Remote Australia	Retrospective Cohort Study	*J. Clin. Sleep Med.*	200	All regional and remote indigenous Australians accessed diagnostic sleep studies at a rate less than Australian general population overall, with a higher likelihood of having a positive diagnostic test.	Residing in a remote community contributes to the difficulty in accessing specialized sleep services.

## Data Availability

Not applicable.

## Supplementary Materials

The following supporting information can be downloaded at: https://www.mdpi.com/article/10.3390/ijerph23050614/s1, File S1: PRISMA Checklist [36].

## Abbreviations

The following abbreviations are used in this manuscript:

PRISMA	Preferred Reporting Items for Systematic Reviews and Meta-Analyses
PICO	Patient/Population, Intervention, Comparison/Control, Outcome
MeSH	Medical Subject Headings
IRB	Institutional Review Board
OSA	Obstructive Sleep Apnea
CPAP	Continuous Positive Airway Pressure
